# Maintenance With Hypomethylating Agents After Allogeneic Stem Cell Transplantation in Acute Myeloid Leukemia and Myelodysplastic Syndrome: A Systematic Review and Meta-Analysis

**DOI:** 10.3389/fmed.2022.801632

**Published:** 2022-02-15

**Authors:** Smith Kungwankiattichai, Ben Ponvilawan, Claudie Roy, Pattaraporn Tunsing, Florian Kuchenbauer, Weerapat Owattanapanich

**Affiliations:** ^1^Division of Hematology, Department of Medicine, Faculty of Medicine Siriraj Hospital, Mahidol University, Bangkok, Thailand; ^2^Department of Pharmacology, Faculty of Medicine Siriraj Hospital, Mahidol University, Bangkok, Thailand; ^3^Vancouver General Hospital, L/BMT Program of British Columbia, Vancouver, BC, Canada; ^4^British Columbia Research Centre, Terry Fox Laboratory, Vancouver, BC, Canada

**Keywords:** acute myeloid leukemia, azacitidine, decitabine, hypomethylating agent, maintenance, transplant

## Abstract

**Introduction:**

Hypomethylating agents (HMAs) seem to have a range of properties favorable to post-allogeneic hematopoietic stem cell transplantation (allo-SCT) maintenance in acute myeloid leukemia (AML) patients.

**Materials and Methods:**

The Embase, MEDLINE, and Cochrane Central Register of Controlled Trials databases were independently searched by two investigators to identify relevant studies published inception to 18 November 2021. These trials compared HMA maintenance to observation following allo-SCT for AML or myelodysplastic syndrome.

**Results:**

The meta-analysis eligibility criteria were fulfilled by 14 studies. The overall survival and relapse-free survival of the HMA maintenance group were superior to the observation group, with a pooled risk ratio (RR) of 1.38 and 1.46, respectively. Moreover, the cumulative incidence of relapse was significantly lower in those who received HMAs. The HMA group also had lower non-relapse mortality compared with the observation group. Overall, the incidences of grades III–IV acute graft-vs.-host disease (GVHD) and chronic GVHD did not differ in both groups. However, when looking specifically at those receiving decitabine maintenance, the rate of chronic GVHD seemed to be lower compared with observation alone.

**Conclusions:**

The current systematic review and meta-analysis illustrated that AML and MDS patients receiving HMA maintenance after allo-SCT had better outcomes in regards to OS, RFS, NRM, CIR as well as a reduced incidence of chronic GVHD.

## Key Messages

- Role of HMAs Maintenance After Allo-SCT in AML Has Been Extensively Studied in Recent Years in Order to Improve Clinical Outcome.
- This Meta-Analysis Demonstrated Favorable Outcome of HMAs Maintenance in Term of Relapse Rate, non-Relapse Mortality, Relapse-Free Survival and Overall Survival.
- Decitabine Maintenance Resulted in Lower Chronic GVHD Rate Compared With Observation Strategy.

## Introduction

Allogeneic stem cell transplantation is the mainstay treatment for AML stratified as intermediate or unfavorable risk as well as for high-risk MDS. This therapy has demonstrated superior efficacy over non-alloSCT approaches in regards to long-term clinical outcomes (1, 2). Nevertheless, even after allo-SCT, 35–45% of patients suffer from disease relapse, leading to dismal outcomes (3, 4).

Several strategies have been adopted to prolong disease-free survival. Based on the time of intervention, they can be categorized into either preemptive approaches—those commenced at the time of detection of minimal residual disease (MRD)—or prophylactic approaches—those initiated in the absence of detectable leukemia. In the case of the prophylactic approaches, both cellular and pharmacological maintenance strategies have been reported, including prophylactic donor leukocyte infusion (DLI), hypomethylating agents (HMAs), histone deacetylase inhibitors, FMS-like tyrosine kinase 3 (FLT3) inhibitors, or isocitrate dehydrogenase inhibitors (5–7). Notably, HMAs have generated considerable research interest in recent years due to their favorable side effect profile.

HMAs exhibit several properties that make them suitable for post-allo-SCT maintenance. They mediate a direct anti-leukemic effect in AML and MDS, regardless of their molecular mutation profile. Moreover, their abilities to induce a CD8+ tumor-specific T cell response, together with the expansion of regulatory T cells, lead to an epigenetically enhanced Graft vs. Leukemia (GVL) effect that is not counterbalanced by an increased risk of GVHD (8–12). Lastly, they are safe and well-tolerated by AML patients in remission (13).

Many studies have examined the use of azacitidine and decitabine as maintenance after allo-SCT for AML and MDS. Although the majority of the studies supported consideration of HMA maintenance therapy, the remainder did not demonstrate clear benefits (14–29). A recent systematic review explored the safety and efficacy of maintenance treatment following allo-SCT in AML and MDS. It demonstrated rates of 65.6 and 56.2% for the 2-year overall survival (OS) and the relapse-free survival (RFS), respectively, of HMA-treated patients. In addition, acute and chronic GVHD were found in 39.9 and 44.4%, of patients respectively. These results suggest that HMA maintenance could be employed to prolong RFS and OS (30). Nonetheless, the benefit of HMA maintenance after allo-SCT is still uncertain.

This meta-analysis was performed to review all relevant studies to compare the outcomes of patients undergoing allo-SCT for AML or MDS receiving HMA maintenance therapy with observation only.

## Materials and Methods

### Data Sources and Searches

The Embase, MEDLINE, and Cochrane Central Register of Controlled Trials databases were independently searched by two investigators (B.P., W.O.) to identify relevant studies published from inception to November 18, 2021. The search terms consisted of words associated with HMAs, acute myeloid leukemia, myelodysplastic syndrome, and stem cell transplantation. Supplementary Data 1 details the exhaustive search strategy lists. The study was conducted in accordance with the PRISMA (Preferred Reporting Items for Systematic Reviews and Meta-Analysis) guidelines (Supplementary Data 2).

### Selection Criteria and Data Extraction

The inclusion criteria were as follows: (1) studies had to be either randomized controlled trials (RCTs) or cohort studies (prospective or retrospective); (2) the patients underwent allo-SCT for AML or MDS; (3) the studies compared two patient groups: one receiving an HMA post-allo-SCT, and the other being an observational group; and (4) the studies needed to report at least one of our primary outcomes of interest (OS, RFS, grades III–IV acute GVHD, and chronic GVHD). The secondary outcomes of interest were the CIR and NRM. Study eligibility was individually assessed by three investigators (B.P., W.O, S.K.); disagreements were resolved by consensus.

Two investigators (B.P., W.O.) utilized a standardized collection form to extract the baseline characteristic data of the patients in each group, along with details of the primary and secondary outcomes of interest. The extracted data was cross-checked to confirm its accuracy.

### Definitions of Outcomes

The OS rate was defined as the time between the stem cell infusion and the time of death or last follow-up, while RFS was defined as the time interval from the stem cell infusion to the date of relapse or death from any cause. All causes of death (other than death from a relapse) were used to calculate the NRM rate.

### Quality Assessment

Two investigators (B.P., W.O.) assessed the quality of each study using the Jadad scale for RCTs and the Newcastle–Ottawa scale for cohort studies (31, 32).

### Statistical Analysis

The Mantel–Haenszel method was used to combine the effect estimates and 95% confidence intervals (CIs) of each study, and to calculate the pooled odds ratio (OR) with 95% CI (33). A random-effects model was preferred over a fixed-effects model because it was more likely that high heterogeneity would be found among the studies. Statistical heterogeneity was calculated using Cochran's Q test, estimated by the heterogeneity (*I*^2^) statistic. There were four heterogeneity levels: insignificant (*I*^2^ values of 0–25%), low (*I*^2^ values of 26–50%), moderate (*I*^2^ values of 51–75%), and high (*I*^2^ values of > 75%) (34). The presence of a publication bias was visualized by a funnel plot along with Egger's regression test. Due to a lack of clinical studies, a subgroup analysis based on the types of HMAs could not be performed. All statistical analyses were performed using the Review Manager (RevMan) software (version 5.3; The Cochrane Collaboration, Oxford, UK) and “meta” package version 5.1-0. This study was registered at www.inplasy.com as #INPLASY2021110078.

## Results

### Search Results

**Figure 1:**
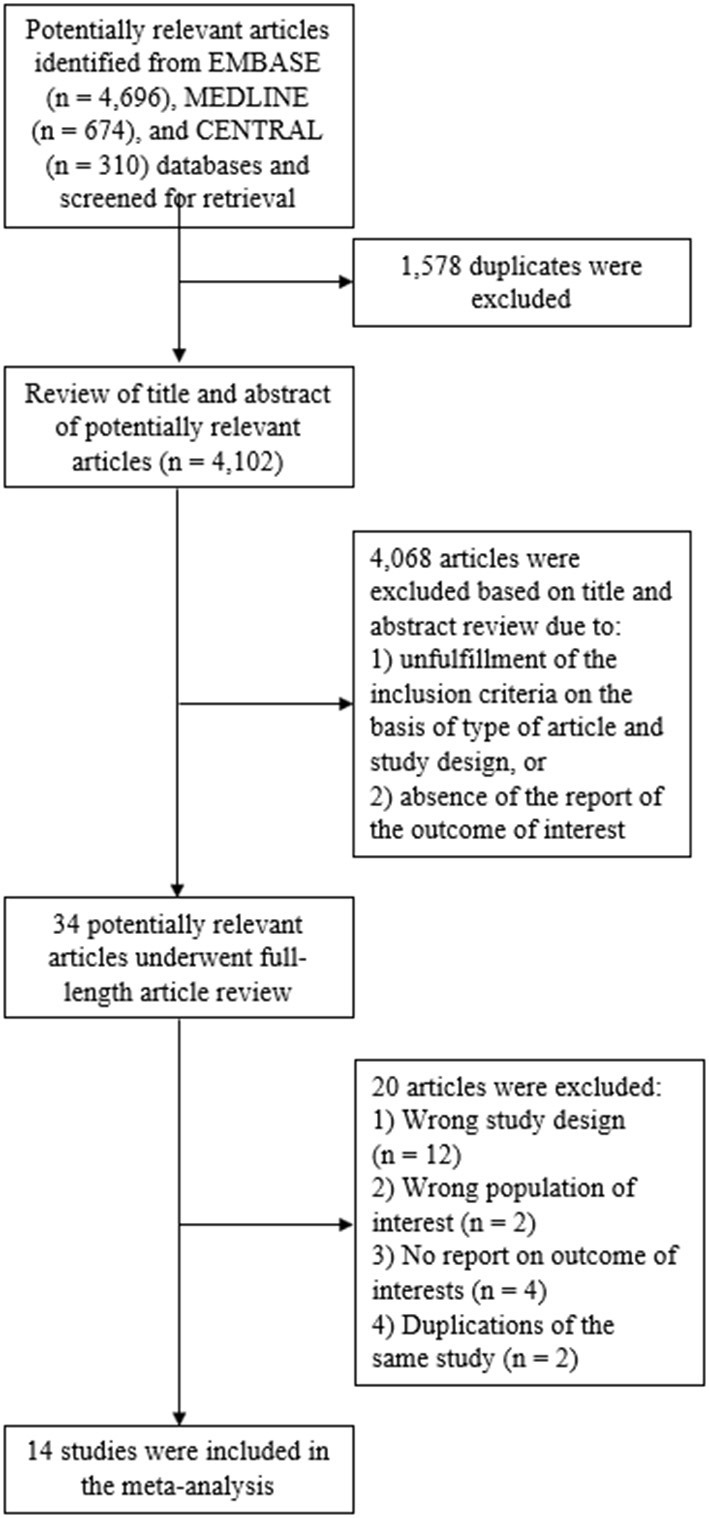
Study identification and literature review process.

The systematic search of the Embase, MEDLINE and Cochrane Central Register of Controlled Trials databases identified 5,680 articles, from which 1,578 duplicates were removed. This resulted in 4,102 articles available for title and abstract review. Subsequently, 4,068 articles were excluded as the article type and study design did not fulfill the inclusion criteria, or there was no report on a primary outcome of interest. The remaining 34 articles underwent full-length review and 20 of those were excluded for the aforementioned reasons. Ultimately, the eligibility criteria for our meta-analysis were met by 14 studies: two RCTs, two prospective cohort study, and 10 retrospective cohort studies (14–22, 25, 27–29). Twelve of these compared azacitidine maintenance to observation, whereas two compared decitabine maintenance to observation. Figure 1 illustrates the full literature review and selection process.

### Baseline Patient Characteristics

The 14 included studies were composed of 533 patients who received HMAs as maintenance, and another 784 patients who were observed post-allo-SCT. The age of the participants varied greatly (HMA group: 2 to 78 years; and observation group: 2 to 75 years). AML accounted for the largest proportion of the disease subtypes in the HMA group (84.8%), followed by MDS (14.6%) and mixed-phenotype leukemia (0.6%). These values were similar to the corresponding proportions found in the observation group (87.5, 11.2, and 1.3%, respectively). In both groups, matched unrelated donors and matched sibling donors were the most common donor sources for allo-SCT, accounting for 45.2 and 26.8%, respectively. In addition, myeloablative conditioning regimens (68.2%) were used more frequently compared to reduced-intensity conditioning regimens (31.8%). Details of the patient characteristics, such as disease status before allo-SCT, MRD status before allo-SCT cytogenetic risk, prior treatment and HMA before allo-SCT, comorbidities and performance status [Hematopoietic Cell Transplantation-Comorbidity Index (HCT-CI)], study period and quality assessment are summarized in Table 1. Details of the donor types, stem cell source, median dose of CD34+ stem cells, conditioning regimens, GVHD prophylaxis, MRD status after allo-SCT, maintenance protocols and prophylactic DLI are listed in Table 2.

**Table 1:** Patient's baseline characteristics of studies included in the meta-analysis.

**References**	**Group**	**No**.	**Sex (M/F)**	**Median age (years, range)**	**Diseases**	**Cytogenetic risk**	**HCT-CI**	**Disease status before HSCT**	**MRD status before HSCT**	**Hypomethylating agents use before HSCT**	**Study period**	**Type**	**Quality assessment**
Oshikawa (15)	HMA	10	8/2	49.5 (17–60)	AML	High	NA	1: ≥CR2 2: relapse 5: PIF 2: relapse after HSCT	NA	NA	NA	PRO	S: 3 C: 1 O: 3
	Control	30	20/10	50 (18–68)	AML	High	NA	3: ≥CR2 6: relapse 15: PIF 6: relapse after HSCT	NA	NA			
Ovechkina (14)	HMA	58	34/24	28 (2–68)	51: AML 7: MDS	14: High 44: NA	NA	NA	7: MRD+ 51: MRD-	NA	NA	RET	S: 2 C: 2 O: 3
	Control	58	31/27	29 (2–60)	51: AML 7: MDS	10: High 48: NA	NA	NA	5: MRD+ 53: MRD-	NA			
Kaito (16)	HMA	23	NA	54 (17–67)	21: AML 1: MDS 1: MPAL	High	NA	6: CR 17: not in CR	NA	NA	NA	RET	S: 3 C: 2 O: 3
	Control	69	NA	NA	63: AML 3: MDS 3: MPAL	High	NA	NA	NA	NA			
Américo (21)	HMA	17	NA	>18	MDS/ AML	NA	NA	NA	NA	NA	2011–2018	RET	S: 3 C: 1 O: 3
	Control	51	NA	>18	MDS/ AML	NA	NA	NA	NA	NA			
Maples (17)	HMA	25	14/11	56 (24–72)	18: AML 7: MDS	1: Favorable 8: Intermediate 9: High 7: NA	NA	14: CR1 4: CR2 7: MDS	7: MRD+ 18: MRD-	NA	January 2010–December 2016	RET	S: 4 C: 2 O: 3
	Control	50	21/29	54 (20–70)	38: AML 12: MDS	3: Favorable 13: Intermediate 22: High 12: NA	NA	15: CR1 2: CR2 > CR3: 1 12: MDS	13: MRD+ 37: MRD-	NA			
Danylesko (18)	HMA	40	24/16	62 (25–74)	32: AML 8: MDS	21: High 19: Intermediate	NA	19: CR1 4: CR2 7: refractory AML 10: untreated MDS and secondary AML	3: MRD+ 37: NA	NA	NA	RET	S: 2 C: 2 O: 3
	Control	40	NA	NA	NA	NA	NA	NA	NA	NA			
Guillaume (19)	HMA	30	13/17	58 (22–70)	20: AML 10: MDS	15: High 15: NA	NA	16: CR1 6: CR2 5: R/R 3: untreated MDS	NA	4: yes 26: no	November 2011–May 2015	RET	S: 3 C: 2 O: 3
	Control	58	NA	NA	NA	NA	NA	NA	NA	NA			
Joris (20)	HMA	19	NA	52 (18–70)	48: AML 5: MDS	High	NA	21: CR 32: R/R	12: MRD+CR 9: MRD-CR	NA	January 2012–December 2018	RET	S: 3 C: 1 O: 3
	Control	34	NA			NA	NA			NA			
Ali (22)	HMA	59	36/23	62 (23–78)	45: AML 14: MDS	4: Favorable 30: Intermediate 25: High	28: 0 19: 1–2 12: ≥3	38: CR1 4: CR2 3: R/R 14: MDS	9 MRD+ 32 MRD- 18 NA	NA	December 2011–December 2018	RET	S: 3 C: 2 O: 3
	Control	90	57/33	60 (26–73)	76: AML 14: MDS	4: Favorable 37: Intermediate 48: High	33: 0 31: 1–2 26: ≥3	51: CR1 10: CR2 1: CR3 14: R/R 14: MDS	3 MRD+ 30 MRD- 57 NA	NA			
Gao (29)	HMA	100	56/44	30 (3–62)	High-risk AML	7: Favorable 19: Intermediate 74: High	NA	92: CR 1: PR 7: NR	24 MRD+CR 68 MRD-CR	NA	April 2016–January 2017	RCT	R: 1 D: 0 W: 1
	Control	102	61/41	28 (2–52)	High-risk AML	3: Favorable 20: Intermediate 79: High	NA	97: CR 5: NR	29 MRD+CR 68 MRD-CR	NA			
Ma (24)	HMA	21	13/8	28 (10–63)	19: AML 2: MPAL	High	8: 1 9: 2 3: 3 1: ≥4	19: CR1 7: CR2 5: NR	NA	NA	September 2015–October 2018	RET	S: 3 C: 2 O: 3
	Control	63	37/26	29 (8–56)	59: AML 4: MPAL	High	26: 1 24: 2 8: 3 5: ≥4	41: CR1 10: CR2 12: NR	NA	NA			
Oran (25)	HMA	87	51/36	57 (19–72)	65: AML 22: MDS	8: Favorable 33: Intermediate 46: High	28: 0–1 22: 2–3 37: ≥4	54: CR1/2 33: Active disease	NA	NA	April 2009–January 2017	RCT	R: 1 D: 0 W: 1
	Control	94	57/36	57.5 (20–75)	69: AML 25: MDS	15: Favorable 42: Intermediate 37: High	37: 0–1 37: 2–3 20: ≥4	36: CR1/2 58: active disease	NA	NA			
Booth (27)	HMA	13	NA	12.6	AML	High-risk	NA	NA	NA	NA	January 2010–March 2020	RET	S: 3 C: 1 O: 3
	Control	28	NA	7.0	AML	High-risk	NA	NA	NA	NA			
Keruakous (28)	HMA	31	16/15	47	Poor-risk AML	High	NA	31: CR	7 MRD+ 24 MRD-	NA	September 2013–July 2018	PRO	S: 4 C: 2 O: 3
	Control	18	9/9	54	Poor-risk AML	High	NA	18: CR	6 MRD+ 12 MRD-	NA			

*AML, acute myelogenous leukemia; C, compatibility; CR, complete remission; CR1, first complete remission; CR2, second complete remission; CR3, third complete remission; HCT-CI, Hematopoietic Cell Transplantation-Comorbidity Index; HMA, hypomethylating agent; HSCT, hematopoietic stem cell transplantation; M, male; MDS, myelodysplastic syndrome; MPAL, mixed phenotype acute leukemia; MRD, minimal residual disease; NA, not available; No., number; NR, no remission; O, outcome; PIF, primary induction failure; PR, partial remission; PRO, prospective cohort study; R, randomization; RCT, randomized controlled trial; RET, retrospective cohort study; R/R, relapsed/refractory; S, selection*.

**Table 2:** Peri- and post-transplantation information of studies included in the meta-analysis.

**References**	**Group**	**No**.	**Donor type**	**Stem cell source**	**Median dose of CD34(x10^**6**^/kg)**	**Conditioning regimen (MAC/RIC)**	**GVHD prophylaxis**	**MRD status after HSCT and timing**	**Maintenance therapy after HSCT**	**Prophylactic DLI**
Oshikawa (15)	HMA	10	1: MSD 4: MUD 5: Haplo	6: PB 4: BM	NA	MAC (4: Bu/Cy, 1: Flu/Bu/ATG/TBI 2: Flu/Cy/AraC/ATG/TBI)/ RIC (3:Flu/Mel/ATG/TBI)	MTX/CSA or MTX/TAC	NA	- AZA 30 mg/m^2^ x 7 days combined with GO 3 mg/m2 on Day 8 up to 4 cycles	-
	Control	30	NA	NA	NA	NA		NA	Observation	-
Ovechkina (14)	HMA	58	12: MSD 35: MUD/ MMUD 11: Haplo	NA	NA	16/42	NA	NA	- AZA 35 mg/m^2^ x 5 days every 28 days - Median: 2.5 cycles (1–8 cycles) - Median time of starting AZA: 253 days (27–861 days) after HSCT	36% (21 patients)
	Control	58	22: MSD 28: MUD/ MMUD 8: Haplo	NA	NA	17/41	NA	NA	Observation	–
Kaito (16)	HMA	23	15: Matched donor 8: Haplo	NA	NA	12/11	NA	NA	- AZA 30 mg/m^2^ x 7 days combined with GO 3 mg/m^2^ on Day 8 up to 4 cycles - Median time of starting AZA: 78 days (24–251 days) after HSCT	–
	Control	69	NA	NA	NA	NA	NA	NA	Observation	–
Américo (21)	HMA	17	NA	NA	NA	NA	NA	NA	AZA	-
	Control	51	NA	NA	NA	NA	NA	NA	observation	-
Maples (17)	HMA	25	6: MSD 16: MUD 2: MMUD 1: UCB	21: PB 3: BM 1: CB	NA	23/2 (MAC: 11 Bu/Cy, 11 Bu/Flu, 1 Cy/TBI; RIC: 2 Bu/Flu)	13: TAC/MTX 8: TAC/MMF 4: CSA/MTX	NA	- AZA 32 mg/m^2^ x 5 days every 28 days for 4–6 cycles - Median time of starting AZA: 75 days (42–131 days) after HSCT	–
	Control	50	15: MSD 24: MUD 10: MMUD 1: UCB	43: PB 6: BM 1: CB	NA	47/3 (MAC: 19 Bu/Cy, 15 Bu/Fly, 13 Cy/TBI; RIC: 1 Bu/Flu, 2 ATG/TBI)	32: TAC/MTX 5: TAC/MMF 13: CSA/MTX	NA	Observation	–
Danylesko (18)	HMA	40	8: MSD 32: MUD	NA	NA	20/20	NA	8: MRD + 32: MRD- Timing: NA	- AZA 32–50 mg/m^2^ x 5 days every 28 days for 2 years - Median time of starting AZA: 2.2 months (1.2–6.9 months) after HSCT	8% (3 patients)
	Control	40	NA	NA	NA	NA	NA	NA	Observation	–
Guillaume (19)	HMA	30	13: MSD 15: MUD 2: MMUD	NA	NA	12/18	NA	NA	- AZA 32 mg/m^2^ x 5 days every 28 days for 1 year starting after 8 weeks of HSCT	Start after 3 cycles every 8 weeks of AZA, dose 1–50 x 10^6^/kg of CD3^+^ cells
	Control	58	NA	NA	NA	NA	NA	NA	Observation	–
Joris (20)	HMA	19	13: MSD 27: MUD 4: MMUD 9: Haplo	48: PB 5: BM	7.9	0/53 (sequential RIC)	CSA/MMF	NA	- AZA 37.5 mg/m^2^ x 5 days every 28 days for 1 year	3 cycles of DLI alternating with AZA
	Control	34						NA	Observation	–
Ali (22)	HMA	59	11: MSD 32: MUD 12: Haplo 4: UCB	50: PB 5: BM 4: CB	5.4	18/41	33: CSA/MTX 20: CSA/MMF±PTCy 6: Others	1: MRD + 45: MRD- 13: NA Timing: day +100	- AZA 16–50 mg/m^2^ x 5 days every 28 days for at least 1 cycle (1–22 cycles) - Median time of starting AZA: 62 days (34–236 days) after HSCT	7% (4 patients)
	Control	90	21: MSD 58: MUD 2: Haplo 9: UCB	76: PB 5: BM 9: CB	5.4	42/48	71: CSA/MTX 16: CSA/MMF±PTCy 3: Others	6: MRD + 37: MRD- 47: NA Timing: day +100	Observation	–
Gao (29)	HMA	100	20: MSD 5: MUD 75: Haplo	NA	8.2	100/0	NA	NA	- DAC 5 mg/m^2^ x 5 days every 6–8 weeks up to 6 cycles combining with G-CSF 100 mcg/m^2^ on Day 0–5 of DAC	–
	Control	102	16: MSD 13: MUD 73: Haplo	NA	8.3	102/0	NA	NA	Observation	–
Ma (24)	HMA	21	2: MSD 3: MUD 16: Haplo	5: PB 2: BM 14: PB+BM	NA	21/0	NA	NA	- DAC 20 mg/m^2^ x 5 days every 12 weeks for 4–6 cycles - Median numbers of cycles: 3 (2–8) - Median time of starting DAC: 154 days (55–358 days) after HSCT	–
	Control	63	17: MSD 5: MUD 41: Haplo	20: PB 9: BM 34: PB+BM	NA	63/0	NA	NA	Observation	–
Oran (25)	HMA	87	33: MSD 44: MUD 4: Haplo 6: NA	55: PB 31: BM 1: CB	NA	73/14	4: PTCy 82: TAC/MTX 1: TAC/MMF	NA	- AZA 32 mg/m^2^ x 5 days every 28 days for 12 cycles - Median time of starting AZA: 62 days (42–100 days) after HSCT	–
	Control	94	31: MSD 53: MUD 5: Haplo 4: NA	60: PB 32: BM 2: CB	NA	75/18	9: PTCy 82: TAC/MTX 3: TAC/MMF	NA	Observation	–
Booth (27)	HMA	13	2: MSD 6: MUD 1:MMUD 4: Haplo	NA	NA	12/1	NA	NA	AZA x 6 cycles starting on day+60	DLI x 3 doses after day +120
	Control	28	8: MSD 9: MUD 9:MMUD 2:Haplo	NA	NA	25/3	NA	NA	Observation	
Keruakous (28)	HMA	31	6: MSD 23: MUD 2: Haplo	PB:11 BM:19 NA:1	3.64	24/7	NA	NA	- AZA 32 mg/m^2^ x 5 days every 28 days for 4 cycles starting after 8 weeks of HSCT	–
	Control	18	6: MSD 10: MUD 2: Haplo	PB:11 BM:6 NA:1	3.55	8/10	NA	NA	Observation	–

*ATG, antithymocyte globulin; AZA, azacitidine; BM, bone marrow; BU, busulfan; CSA, cyclosporin A; Cy, cyclophosphamide; DAC, decitabine; DLI, donor lymphocyte infusion; Flu, fludarabine; G-CSF, granulocyte colony-stimulating factor; GO, gemtuzumab ozogamicin; Haplo-, haploidentical; HMA, hypomethylating agent; HSCT, hematopoietic stem cell transplantation; MAC, myeloablative conditioning; MEL, melphalan; MMF, mycophenolate mofetil; MMUD, mismatched unrelated donor; MRD, minimal residual disease; MSD, matched sibling donor; MTX, methotrexate; MUD, match unrelated donor; NA, not available; No., number; PB, peripheral blood; PTCy, post-transplant cyclophosphamide; RIC, reduced intensity consolidation; TAC, tacrolimus; TBI, total body irradiation; UCB, umbilical cord blood*.

### HMA Maintenance Protocols After Allo-SCT

The median time of commencement of the HMAs varied between 56 and 154 days after allo-SCT. Twelve studies used azacitidine (14–22, 25, 27, 28), while two used decitabine (24, 29). The azacitidine dosage was 16–50 mg/m^2^ on Day 1 to Day 5 every 4 weeks for 1–22 cycles. A study from Oshikawa et al. and Kaito et al. combined azacitidine with gemtuzumab ozogamicin for the maintenance protocol (15, 16). Decitabine was administered at 5–20 mg/m^2^ on Day 1 to Day 5 every 6–12 weeks for 1–22 cycles. Some patients received prophylactic DLI in addition to HMA maintenance in five studies (14, 18–20, 22, 27).

### Comparison of Clinical Outcomes of HMA and Observation Groups

**Figure 2:**
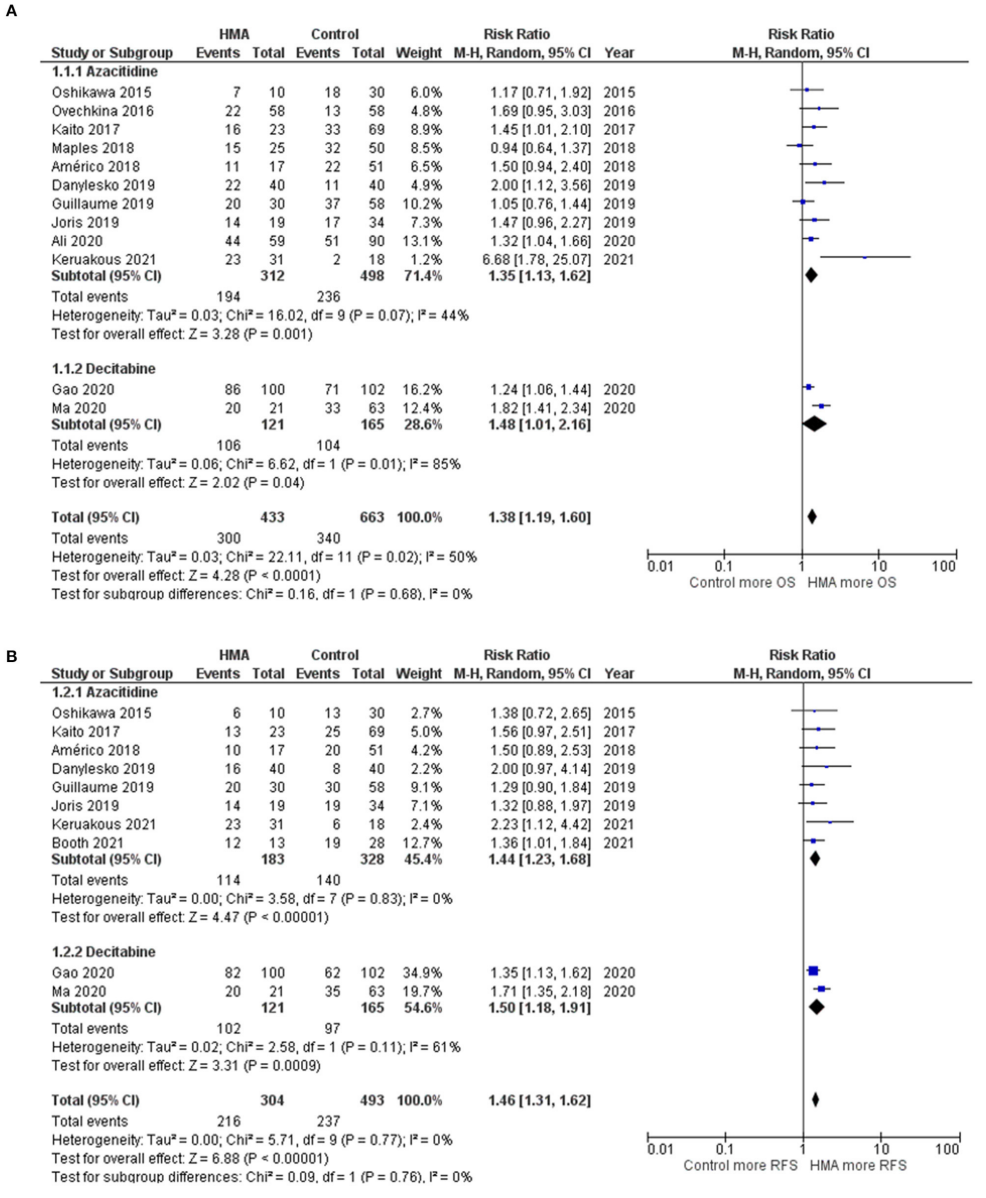
Forest plots of the meta-analysis of HMA maintenance compared with no HMA maintenance. **(A)** OS rate. **(B)** RFS rate.

**Figure 3:**
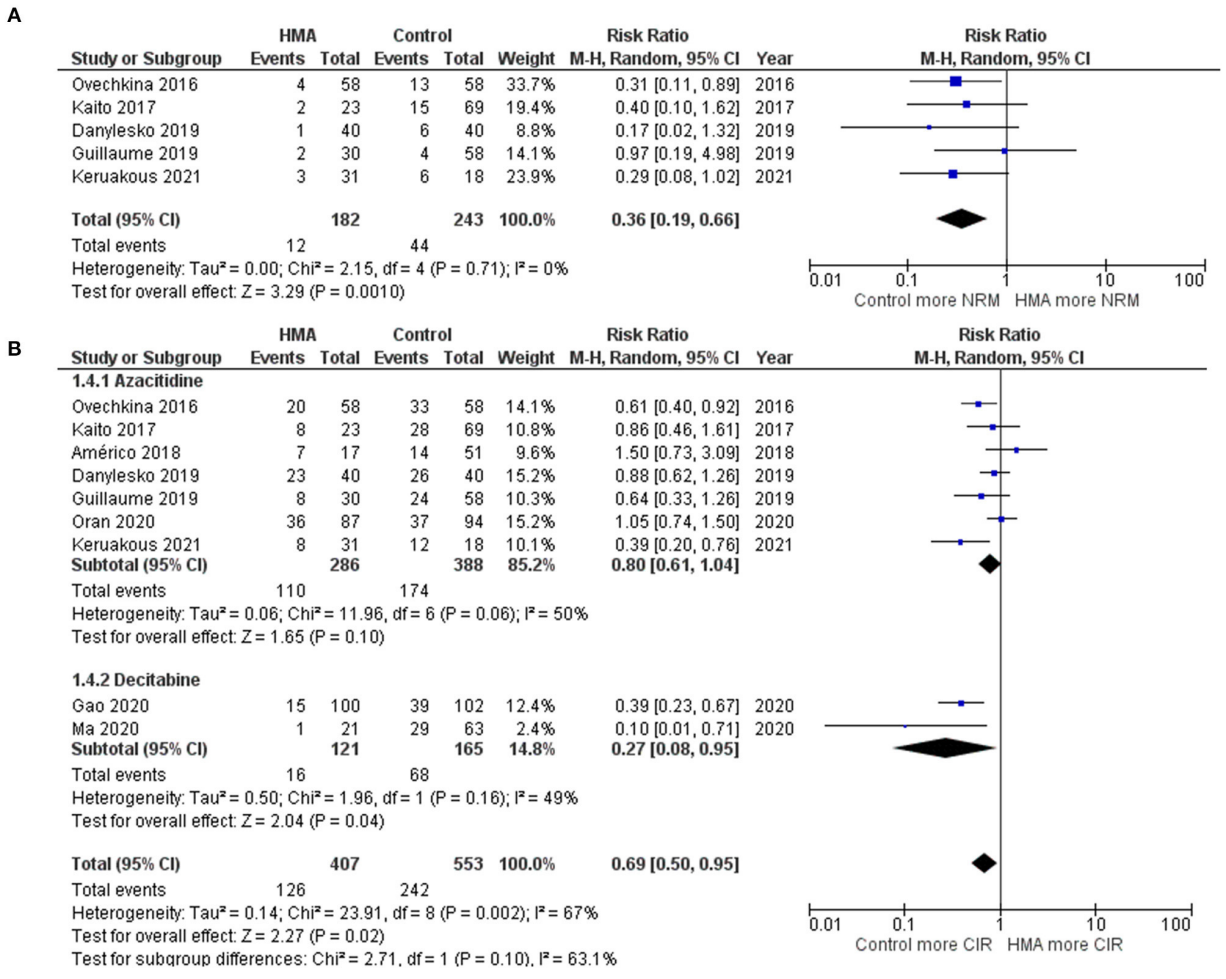
Forest plots of the meta-analysis of HMA maintenance compared with no HMA maintenance. **(A)** NRM rate. **(B)** CIR rate.

**Figure 4:**
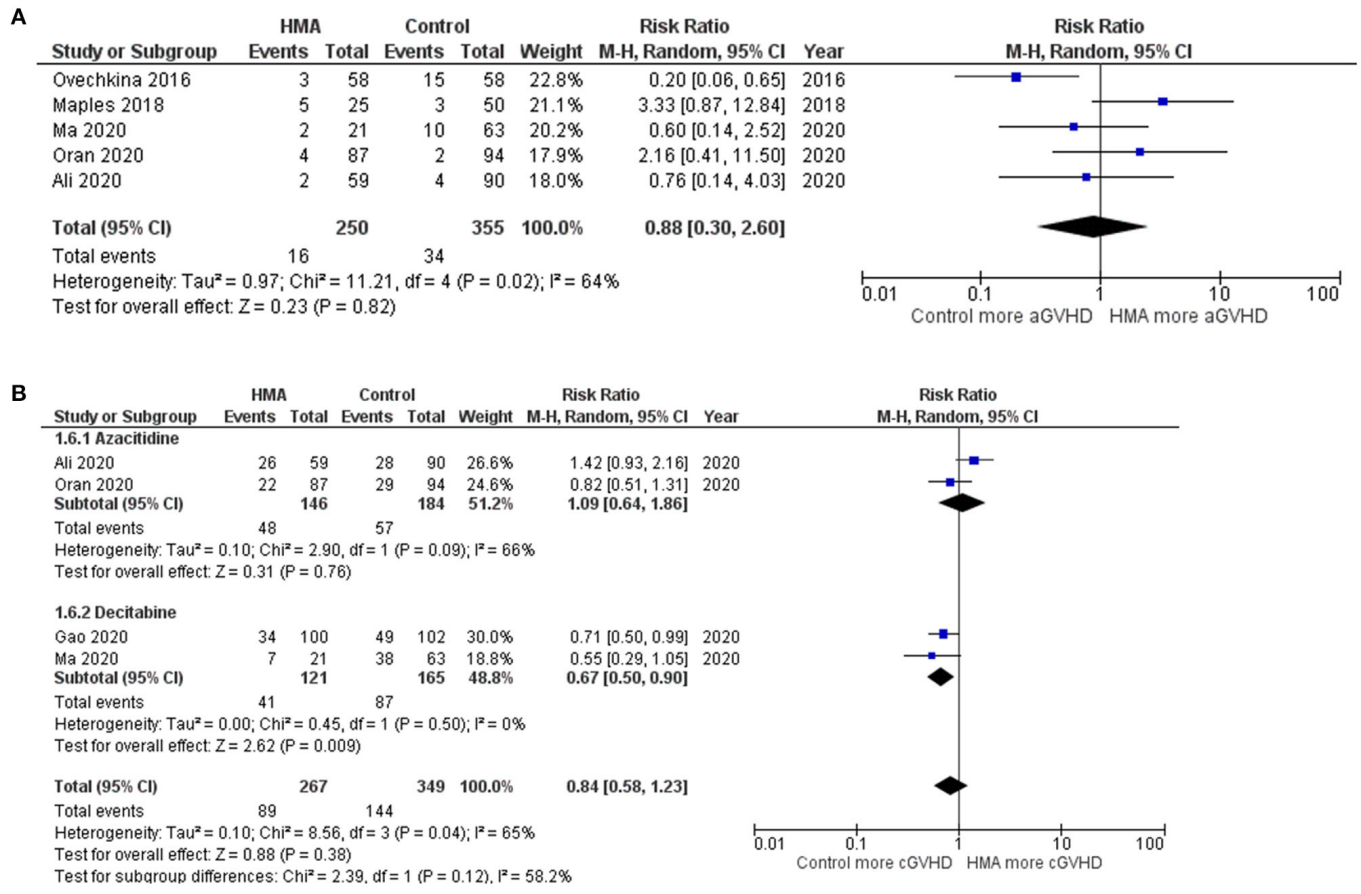
Forest plots of the meta-analysis of HMA maintenance compared with no HMA maintenance. **(A)** grade III–IV aGVHD rate. **(B)** cGVHD rate.

The OS rates were reported as a 1-year rate in three studies (15–17), a 2-year rate in six studies (19–22, 28, 29), and a 3-year rate in three studies (14, 18, 24). The RFS rates were reported as a 1-year rate in two studies (15, 16), a 2-year rate in five studies (19–21, 27, 29), and a 3-year rate in two studies (18, 24). The OS of the HMA group was superior to that of the observation group, with a pooled RR of 1.38 (95% CI, 1.19–1.60; *I*^2^, 50%; Figure 2A) (14–22, 24, 28, 29). Similarly, a pooled meta-analysis found that the RFS was significantly better in patients who received HMAs, with a pooled RR of 1.46 (95% CI, 1.31–1.62; *I*^2^, 0%; Figure 2B) (15, 16, 18–21, 24, 27, 29). The patients receiving HMAs also had a lower NRM than those under observation (pooled RR, 0.36; 95% CI, 0.19–0.66; *I*^2^, 0%; Figure 3A) (14, 16, 18, 19, 28). Furthermore, the CIR was significantly higher for the observed patients (pooled RR, 0.69; 95% CI, 0.50–0.95; *I*^2^, 67%; Figure 3B) (14, 16, 18, 19, 21, 24, 25, 28, 29). However, the incidences of grades III–IV acute GVHD and chronic GVHD of the groups did not differ [pooled RR, 0.88; 95% CI, 0.30–2.60; *I*^2^, 604% (14, 17, 22, 24, 25); and pooled RR, 0.84; 95% CI, 0.58–1.23; *I*^2^, 65% (22, 24, 25, 29); Figures 4A,B, respectively].

Because two studies combined gemtuzumab ozogamicin with HMA and five studies combined DLI with HMA, the efficacy of the HMAs was confirmed by conducting a sensitivity analysis that excluded those seven studies. As with the results of the full analysis, the OS, RFS, and NRM were found to be better for the HMA group than the observation group, whereas the CIR and the incidence of acute GVHD did not differ between the two groups (Supplementary Data 3). Nonetheless, the patients who received HMAs had a significantly lower incidence of chronic GVHD (pooled RR, 0.71; 95% CI, 0.55–0.91; *I*^2^, 0%) (24, 25, 29).

**Figure 5:**
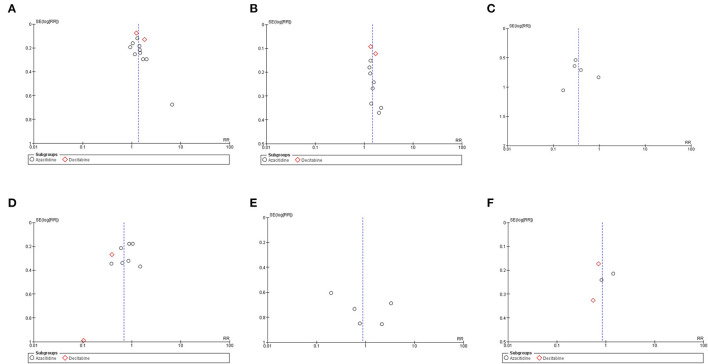
Funnel plots of the meta-analysis of HMA maintenance compared with no HMA maintenance. **(A)** OS rate. **(B)** RFS rate. **(C)** NRM rate. **(D)** CIR rate. **(E)** grade III–IV aGVHD rate. **(F)** cGVHD rate.

Funnel plots of the OS, RFS, NRM, CIR, grades III–IV acute GVHD, and chronic GVHD outcomes of the HMA and observation groups did not show a publication bias (Figure 5). Egger's regression test confirmed this (*p* = 0.1590, 0.2713, 0.8865, 0.1804, 0.3706, 0.8302 for OS, RFS, NRM, CIR, grades III–IV acute GVHD, and chronic GVHD; respectively).

### Subgroup Analysis Based on Study Design

A subgroup analysis based on the study design was performed. There is a trend for a significantly lower risk of chronic GVHD in randomized studies but not in observational studies. The CIR results appear to be similar between randomized studies and observational studies. In observational studies, other parameters which included OS, RFS were significantly better in HMAs arm. However, these parameters could not be analyzed in randomized studies subgroup due to limited number of studies (Supplementary Data 4).

### Subgroup Analysis Based on Each HMA

Two HMAs were used in this meta-analysis: azacitidine and decitabine. The OS, RFS and NRM outcomes of the azacitidine group were significantly better than those of the observation group (Figures 2, 3) (14, 16–20, 22, 28). Likewise, the decitabine arm had superior OS, RFS and CIR outcomes to those of the observation arm (Figures 2, 3) (21, 24, 29). The incidence of grades III–IV acute GVHD and chronic GVHD in patients who received azacitidine were similar to those under observation (Figure 4) (14, 16, 22, 25). Interestingly, the rate of chronic GVHD in the decitabine group was significantly lower than in the observation group (pooled RR, 0.67; 95% CI, 0.50–0.90; *I*^2^, 0%; Figure 4B) (24, 29).

### Subgroup Analysis of Patients Who Received HMAs in Combination With DLI

**Figure 6:**
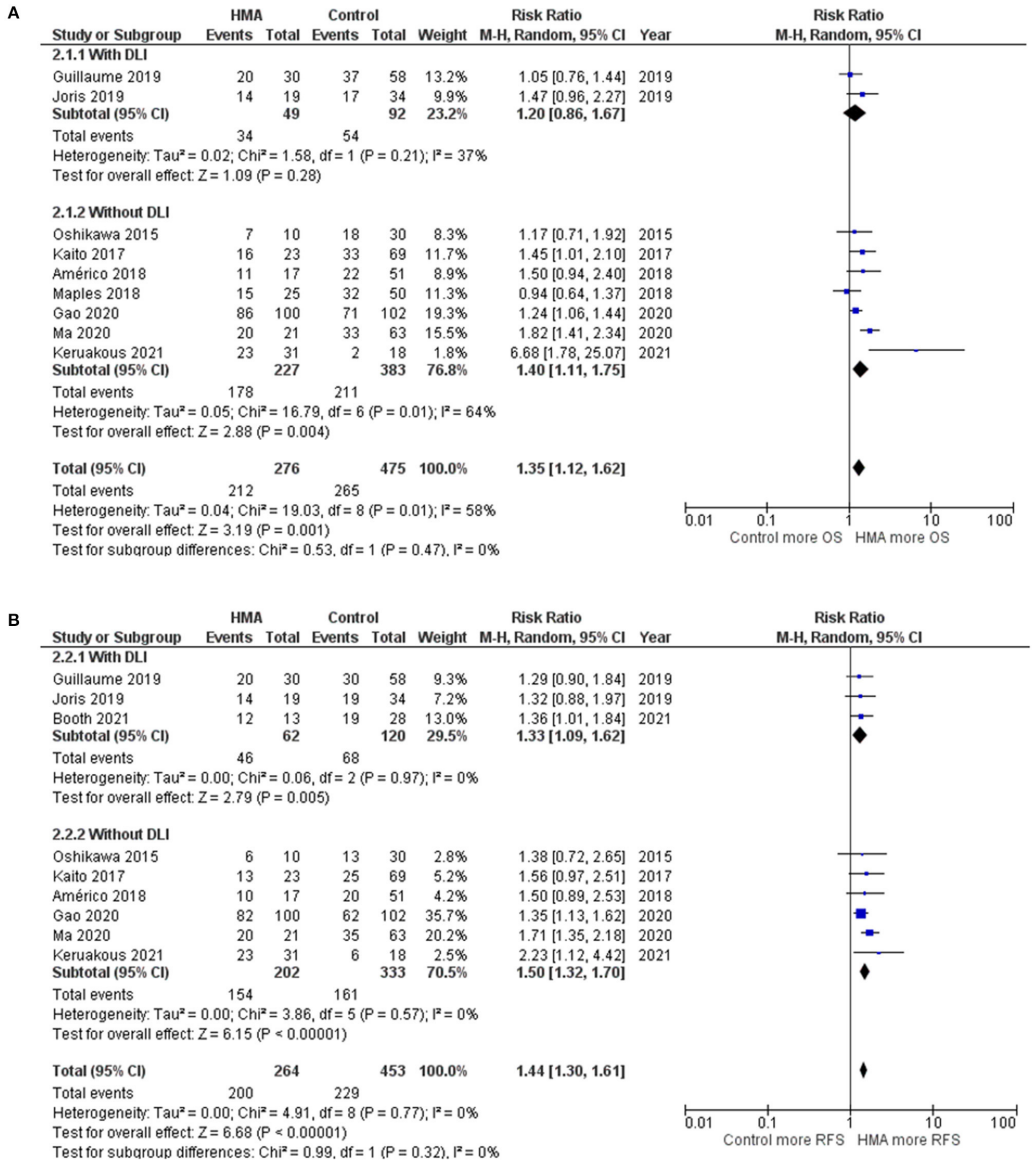
Subgroup analysis of studies with patients receiving HMA maintenance with DLI and without DLI **(A)** OS rate **(B)** RFS rate.

The work by Guillaume et al., Joris et al., and Booth et al. compared HMAs in combination with DLI and observation arms. Although the OS and RFS outcomes tended to be better for HMAs combined with DLI, only RFS rates were statistically significant (pooled RR, 1.20; 95% CI, 0.86–1.67; *I*^2^, 37%; and pooled RR, 1.33; 95% CI, 1.09–1.62; *I*^2^, 0%; Figure 6) (19, 20, 27).

### Sensitivity Analyses

In total, three sensitivity analyses were conducted. The first analysis was performed on studies which recruited only patients with AML. Only RFS and CIR were significantly better in HMAs arm. In addition, there was a trend of superior OS in HMAs group. Nevertheless, other parameters could not be evaluated due to limited number of studies (Supplementary Data 5). The second analysis was performed on studies with adult patients. The OS, RFS and NRM were significantly superior in HMAs arm while the other outcomes did not show significant superiority (Supplementary Data 6). The third analysis was performed on studies with low risk of bias by selecting only studies with a Newcastle-Ottawa scale of at least eight or Jadad score of at least two. Similar results were obtained compared to the main results (Supplementary Data 7).

### Quality of Evidence Using Grading of Recommendations Assessment, Development, and Evaluation Approach

The quality of evidence generated by the current systematic review and meta-analysis is moderate.

## Discussion

An earlier systemic review that focused on the safety and efficacy of HMAs as post-allo-SCT maintenance for AML and MDS found acceptable OS and RFS rates without a heightened GVHD rate (30). Unfortunately, a detailed analysis of the clinical outcomes of the control and treatment groups was not reported. A recent meta-analysis of HMAs and FLT3 Inhibitors as maintenance treatment for AML and MDS after allo-SCT showed a high percentage of OS and RFS (35). Due to the available limited studies comparing clinical outcomes between HMAs and observation arms, we focused on comparing the benefits of HMA maintenance following allo-SCTs with an observation approach and further analyzed the efficacy in each HMA subgroup. Notably, OS, RFS, NRM, and CIR were markedly improved with HMA maintenance. Our subgroup analysis demonstrated the advantages of both azacitidine and decitabine use in this setting. In terms of safety, the HMAs were not associated with a higher GVHD incidence. In the case of decitabine for post allo-SCT maintenance, the rate of chronic GVHD seemed to be lower than that of the observation arm. Previous studies showed that HMA maintenance had low rates of toxicities and infectious complications even if the treatment is given to elderly patients (36, 37). Furthermore, a prior study reported that 6.8% of post-allo-SCT patients experienced isolated extramedullary relapse which translate into dismal survival outcome (38). Accordingly, prophylaxis scheme post allo-SCT is a rational option to mitigate either isolated extramedullary or bone marrow relapse risk. Taken together, the use of HMAs is a feasible therapy for AML and MDS patients during the post-allo-SCT period, and it should be offered broadly to post allo-SCT patients.

Recently, an oral formulation of azacitidine (CC-486) was approved by the U.S. Food and Drug Administration for the continued treatment of adult AML. Based on data from the phase 3 QUAZAR AML-0001 clinical trial, the patients must have achieved first complete remission or must have an incomplete blood count recovery following intensive induction chemotherapy, and be unable to complete intensive curative therapy (39). The oral formulation of azacitidine may enhance patient convenience, eliminate injection-site reactions, and facilitate long-term administration. The application of this product in a post-allo-SCT setting has since been verified in a phase I/II study, which supports the promising clinical activity (40). A randomized, phase III trial to validate its efficacy is in development.

Although the present analysis confirms the usefulness of HMAs, several limitations are noted. First, variations in the disease status prior to transplantation, the difference in conditioning regimen, and the treatment and protocols of the studies (HMA dosage, number of cycles, and dates of administration) could lead to a diversity of clinical outcomes. Second, there was missing data on the European Group for Blood and Marrow Transplantation risk score and disease risk index. It is possible that older patients were selected to have less comorbidities, better performance status, and less prior treatment burden compared to the younger ones; which could be the confounding factors behind similar outcomes. Furthermore, the lack of comorbidity is an issue in identifying risk factors for NRM. Third, there is a recent trend in using MRD status in pre- and post-allo-SCT setting to classify MRD-positive patients who would benefit from HMAs maintenance after allo-SCT (41). However, MRD assessment data was scarce in published included trials precluding a subgroup analysis. In addition, the incorporation of other agents, such as gemtuzumab ozogamicin and G-CSF into each treatment protocol also impact outcomes of clinical trials. Large-scale randomized trials are warranted to clarify all of these unresolved issues.

## Conclusion

The current systematic review and meta-analysis illustrated that the patients receiving HMA maintenance post-Allo-SCT had significantly better outcomes with regards to OS, RFS, NRM, and CIR. Furthermore, if decitabine was used for maintenance, the rate of chronic GVHD seemed to be lower than that of the observation arm. Further data, preferably from large prospective studies, is warranted to confirm the benefit of HMA-based maintenance after allo-SCT as well as describe the optimal agent, administration schedule, and the sub-groups of patients who benefit from such intervention.

## Data Availability Statement

The original contributions presented in the study are included in the article/Supplementary Material, further inquiries can be directed to the corresponding authors.

## Ethics Statement

The need for ethics approval by institutional board review was waived as this study did not directly involve human subjects.

## Author Contributions

BP and WO collected the data. BP performed the statistical analyses. WO and SK drafted the manuscript and prepared the final version. FK made critical revisions. All authors designed the study, read, and approved the final manuscript.

## Conflict of Interest

The authors declare that the research was conducted in the absence of any commercial or financial relationships that could be construed as a potential conflict of interest.

## Publisher's Note

All claims expressed in this article are solely those of the authors and do not necessarily represent those of their affiliated organizations, or those of the publisher, the editors and the reviewers. Any product that may be evaluated in this article, or claim that may be made by its manufacturer, is not guaranteed or endorsed by the publisher.
