# Supplementary material for: Maintenance With Hypomethylating Agents After Allogeneic Stem Cell Transplantation in Acute Myeloid Leukemia and Myelodysplastic Syndrome: A Systematic Review and Meta-Analysis
Source: Front Med (Lausanne). 2022 Feb 15;9:801632. doi: 10.3389/fmed.2022.801632 (PMC8887643; doi:10.3389/fmed.2022.801632)
Supplement: Supplementary file 1 [file Data_Sheet_1.docx]

**Supplementary Material 1 -** Search Strategy

**Embase Database**

1. ‘hypomethylating agent’/exp OR ‘hypomethylating agent’
2. ‘azacitidine’/exp OR ‘azacitidine’
3. ‘decitabine’/exp OR ‘decitabine’
4. ‘cc 486’/exp OR ‘cc 486’
5. ‘vidaza’/exp OR vidaza
6. ‘dacogen’/exp OR dacogen
7. ‘myelodysplastic syndrome’/exp OR ‘myelodysplastic syndrome’
8. ‘acute myeloid leukemia’/exp OR ‘acute myeloid leukemia’
9. ‘acute myelogenous leukemia’/exp OR ‘acute myelogenous leukemia’
10. ‘maintenance therapy’/exp OR ‘maintenance therapy’
11. ‘maintenance chemotherapy’/exp OR ‘maintenance chemotherapy’
12. ‘maintenance’/exp OR maintenance
13. ‘transplantation’/exp OR ‘transplantation’
14. ‘transplant’/exp OR ‘transplant’
15. #1 OR #2 OR #3 OR #4 OR #5 OR #6
16. #7 OR #8 OR #9
17. #10 OR #11 OR #12 OR #13 OR #14
18. #15 AND #16 AND #17

**Ovid Medline Database**

1. hypomethylating agent.mp.
2. azacitidine.mp. or exp Azacitidine/
3. decitabine.mp. or exp Decitabine/
4. cc-486.mp.
5. vidaza.mp.
6. dacogen.mp.
7. myelodysplastic syndrome.mp. or exp Myelodysplastic Syndromes/
8. acute myeloid leukemia.mp. or exp Leukemia, Myeloid, Acute/
9. acute myelogenous leukemia.mp.
10. maintenance therapy.mp.
11. maintenance chemotherapy.mp. or exp Maintenance Chemotherapy/
12. exp Maintenance/ or maintenance.mp.
13. exp Transplantation/ or exp Stem Cell Transplantation/ or transplantation.mp. exp Cord Blood Stem Cell Transplantation/ or exp Cell Transplantation/ or exp Bone Transplantation/ or exp Bone Marrow Transplantation/ or exp Peripheral Blood Stem Cell Transplantation/ or exp Hematopoietic Stem Cell Transplantation/
14. transplant.mp. or exp Transplants/
15. 1 or 2 or 3 or 4 or 5 or 6
16. 7 or 8 or 9
17. 10 or 11 or 12 or 13 or 14
18. 15 and 16 and 17

**Cochrane CENTRAL Database**

1. hypomethylatMeSH descriptor: [Azacitidine] explode all trees
2. MeSH descriptor: [Decitabine] explode all trees
3. (hypomethylating agent) OR ("Vidaza") OR (dacogen) OR (cc-486)
4. ("azacitidine") OR ("decitabine")
5. MeSH descriptor: [Myelodysplastic Syndromes] explode all trees
6. MeSH descriptor: [Leukemia, Myeloid, Acute] explode all trees
7. ("acute myelogenous leukemia") OR ("acute myeloid leukemia") OR ("myelodysplastic syndrome")
8. MeSH descriptor: [Maintenance Chemotherapy] explode all trees
9. MeSH descriptor: [Maintenance] explode all trees
10. MeSH descriptor: [Transplantation] explode all trees
11. MeSH descriptor: [Hematopoietic Stem Cell Transplantation] explode all trees
12. ("maintenance therapies") OR ("maintenance therapy") OR ("maintenance") OR ("transplant") OR (transplantation)
13. ("hematopoietic stem cell transplantation") OR ("hematopoietic stem cell transplant")
14. #1 OR #2 OR #3 OR #4
15. #5 OR #6 OR #7
16. #8 OR #9 OR #10 OR #11 OR #12 OR #13
17. #14 AND #15 AND #16
