# Supplementary material for: Maintenance With Hypomethylating Agents After Allogeneic Stem Cell Transplantation in Acute Myeloid Leukemia and Myelodysplastic Syndrome: A Systematic Review and Meta-Analysis
Source: Front Med (Lausanne). 2022 Feb 15;9:801632. doi: 10.3389/fmed.2022.801632 (PMC8887643; doi:10.3389/fmed.2022.801632)
Supplement: Supplementary file 6 [file Data_Sheet_6.docx]

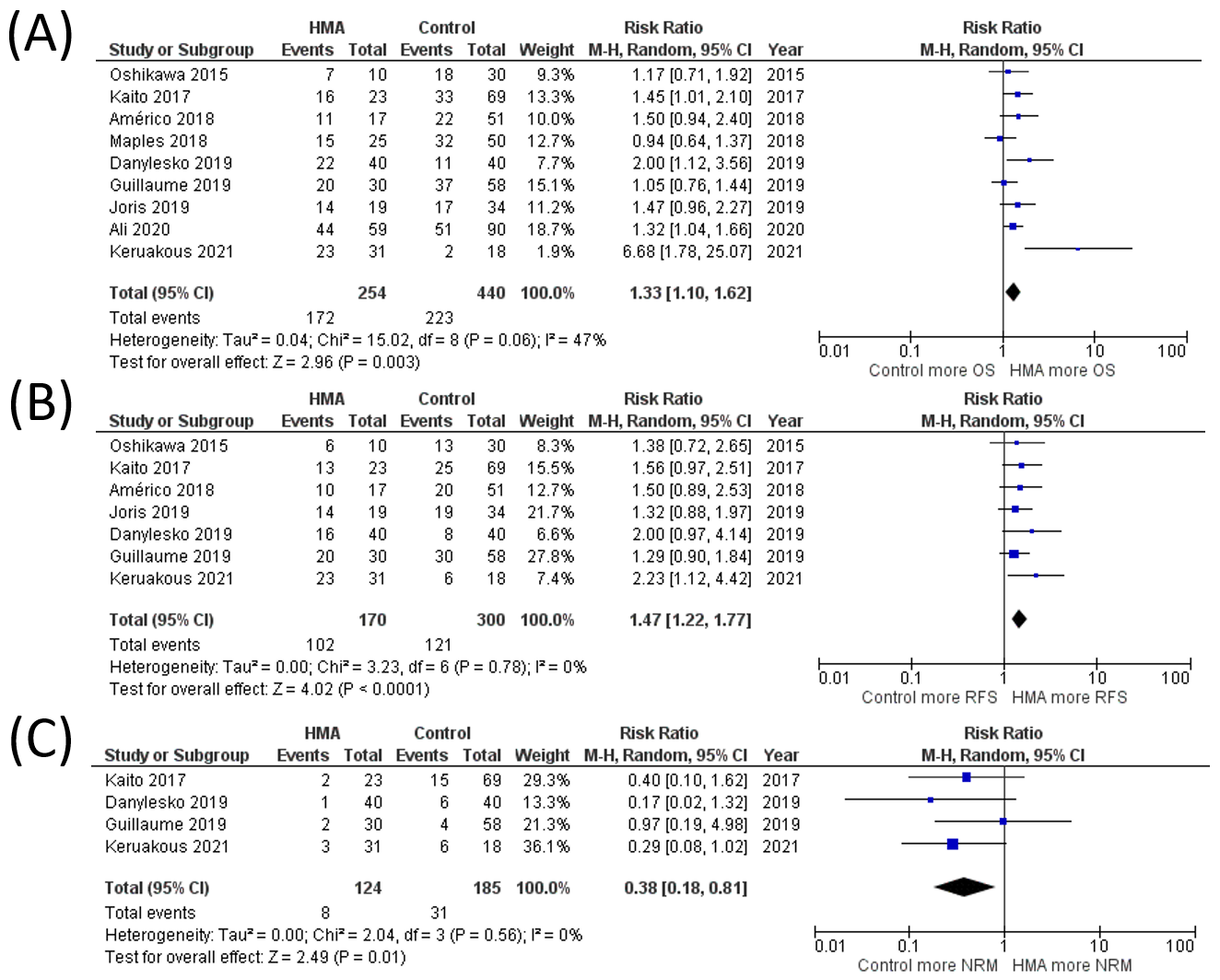


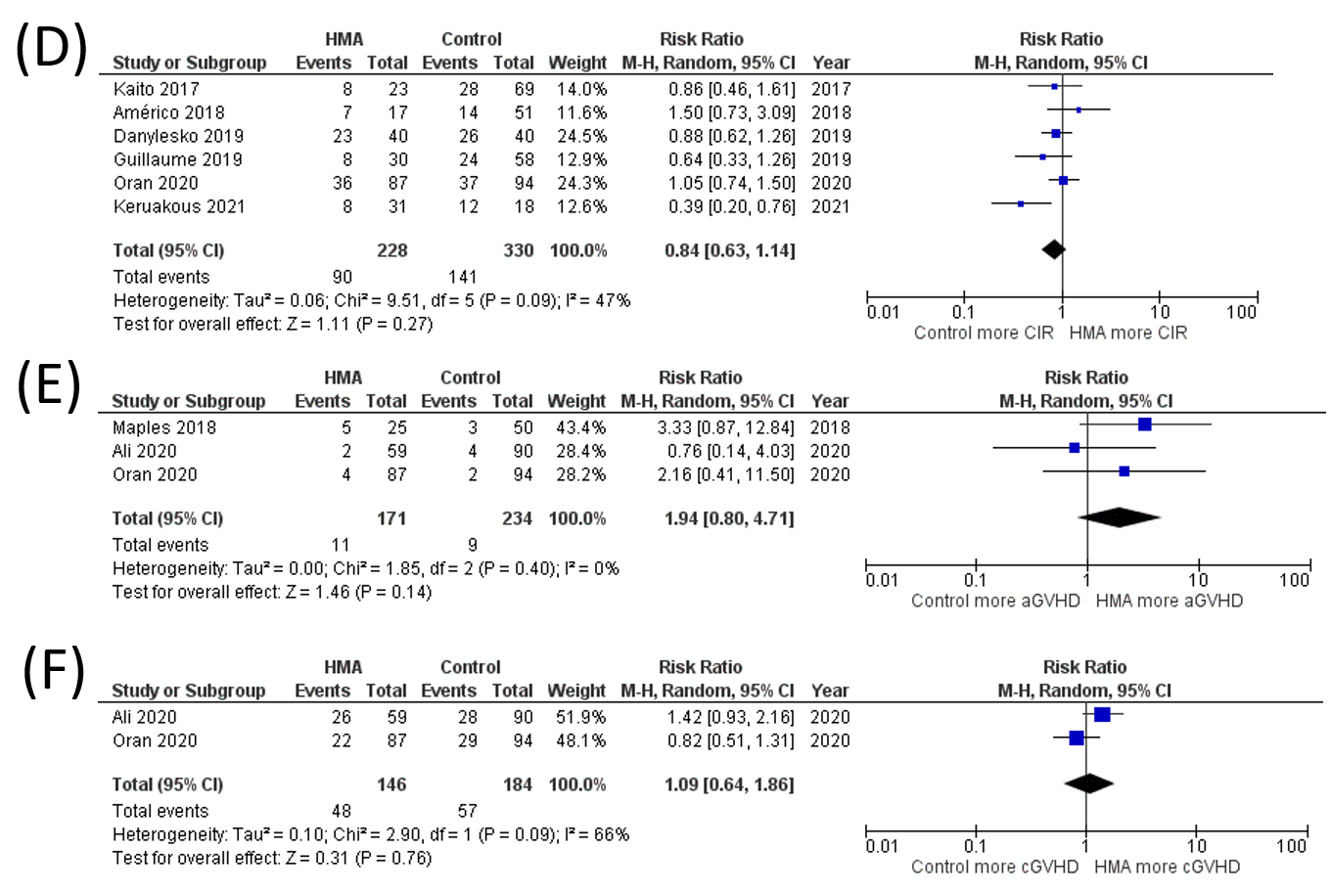


**Supplementary Data 6** Sensitivity analysis of studies with only adult patients with HMA maintenance (A) OS rate (B) RFS rate (C) NRM rate (D) CIR rate (E) grade III-IV aGVHD rate (F) cGVHD rate
